# Novel Molecular Basis for Synapse Formation: Small Non-coding Vault RNA Functions as a Riboregulator of MEK1 to Modulate Synaptogenesis

**DOI:** 10.3389/fnmol.2021.748721

**Published:** 2021-09-24

**Authors:** Shuji Wakatsuki, Toshiyuki Araki

**Affiliations:** Department of Peripheral Nervous System Research, National Institute of Neuroscience, National Center of Neurology and Psychiatry, Tokyo, Japan

**Keywords:** local translation, MAP kinase pathway, MVP (major vault protein), vault complex, synaptogenesis

## Abstract

Small non-coding vault RNAs (vtRNAs) have been described as a component of the vault complex, a hollow-and-barrel-shaped ribonucleoprotein complex found in most eukaryotes. It has been suggested that the function of vtRNAs might not be limited to simply maintaining the structure of the vault complex. Despite the increasing research on vtRNAs, little is known about their physiological functions. Recently, we have shown that murine vtRNA (mvtRNA) up-regulates synaptogenesis by activating the mitogen activated protein kinase (MAPK) signaling pathway. mvtRNA binds to and activates mitogen activated protein kinase 1 (MEK1), and thereby enhances MEK1-mediated extracellular signal-regulated kinase activation. Here, we introduce the regulatory mechanism of MAPK signaling in synaptogenesis by vtRNAs and discuss the possibility as a novel molecular basis for synapse formation.

## Introduction

Non-coding RNAs (ncRNAs) function at the RNA level without being translated into proteins. Representative ncRNAs include ribosomal RNAs and transfer RNAs that are involved in protein synthesis. In recent years, ncRNAs have been reported to regulate many physiological processes, including transcription, translation, RNA processing, and chromatin regulation, and to contribute to protein stability and subcellular localization (Huttenhofer et al., 2005; Amaral et al., 2008; Tuck and Tollervey, 2011; Gebetsberger and Polacek, 2013; Sabin et al., 2013). Among various ncRNAs, small non-coding vault RNAs (vtRNAs) were first described as components of the giant ribonucleoprotein complex, the vault complex. The vault complex is composed of vtRNA and multiple copies of protein molecules, including major vault protein (MVP), and is ubiquitously present in most eukaryotes. There are multiple vtRNA paralogs, and the number varies from species to species: four expressed in humans, i.e., hvtRNA1-1, hvtRNA1-2, hvtRNA1-3, and hvtRNA2-1, and one expressed in mice, i.e., mouse vtRNA (mvtRNA). Previous reports have suggested that vtRNAs have different physiological functions beyond maintaining structural integrity of the vault protein complex (Nandy et al., 2009; Li et al., 2015; Horos et al., 2019).

Establishment of axon/dendrite polarity is an important step in neuronal differentiation (Barnes and Polleux, 2009; Yogev and Shen, 2017). Autism spectrum disorders (ASD), a group of high-prevalence neurodevelopmental disorders, are known to share common cellular/molecular characteristics, including abnormal morphology of synaptic connections, which result in synaptic dysfunction (Mohammad-Rezazadeh et al., 2016). Intracellular signaling, including protein kinases, play a pivotal role in synapse formation and regulation. Among such kinases, the mitogen activated protein kinase (MAPK) signaling pathway that leads to activation of extracellular signal-regulated kinase (ERK) plays an important role in local protein synthesis in dendrites, in the formation and stabilization of dendritic spines, and in the regulation of synaptic plasticity in the brain (Mao and Wang, 2016; Miningou and Blackwell, 2020). Unfortunately, however, their exact regulatory mechanisms remain unclear.

Recently, we showed that mvtRNA promotes synapse formation by activating the MAPK signaling pathway (Wakatsuki et al., 2021a,b). mvtRNA enhances ERK activation by binding to and activating MAPK kinase (MEK). Here we introduce the new role of vtRNAs and discuss their previously unknown roles for synapse formation.

## Mechanism of Regulation of Synapse Formation by Vault RNAs

Aurora-A was originally reported as a kinase that regulates cell division. Subsequently, Aurora-A was also found in non-dividing neurons and has recently been reported to play an important role in the regulation of neuronal morphological differentiation, including neurite outgrowth and polarity formation (Khazaei and Puschel, 2009; Mori et al., 2009; Pollarolo et al., 2011). To clarify the details of Aurora-A dependent processes in neurons, we screened substrates for Aurora-A kinase activity in neurons and found MVP as a prime candidate (Wakatsuki et al., 2021b). We found that the expression level of MVP expression in the brain and the degree of Aurora-A-MVP interaction in the postsynaptic region increase with development and remain stable in maturity. Based on these findings, we considered the possibility that intracellular signals of the Aurora-A-MVP axis are involved in synapse formation and decided to investigate this mechanism in detail.

It has been suggested that MVP interacts with ERK and functions as a scaffold to regulate its signaling (Kolli et al., 2004; Kim et al., 2006; Berger et al., 2009). As mentioned above, the MAPK signaling pathway plays an important role in the regulation of synaptogenesis. Consistent with this, we were able to confirm the presence of activated ERK in the postsynaptic region purified from mouse brain. To investigate the relationship between synaptogenesis through the Aurora-A-MVP pathway and ERK activity, we performed overexpression of Aurora-A or its constitutively active mutant together with MVP in primary cultured cortical neurons, and found that the enhancement of the Aurora-A-MVP signal increased ERK activity and synaptogenesis in neurites. Conversely, knockdown of the *mvp* gene by RNA interference (RNAi) decreased ERK activity and synaptic formation. We also confirmed the functionality of the increased synapses in response to the Aurora-A-MVP signaling by an increase of FM dye uptake into synaptic vesicles and glutamate-stimulated intracellular calcium ion concentrations. These results indicate that enhancement of the Aurora-A-MVP pathway promotes functional synaptogenesis (Wakatsuki et al., 2021b).

It has been suggested that the vault complex is transported through the neurite by a fast transporter (Herrmann et al., 1999; Li et al., 1999). The vault complex binds to several mRNAs that are translated in dendrites, such as tissue plasminogen activator and protein tyrosine phosphatase non-receptor type 5 (Paspalas et al., 2008). These reports strongly suggest that the vault complex serves as an mRNA transporter to neurites. Aurora-A activated in response to neuronal activity has been reported to positively regulate such local translation by phosphorylating cytoplasmic polyadenylation element (CPE) binding protein. The phosphorylated CPE binding protein binds to the CPE-binding sequence of mRNAs in neurites in response to neuronal activity in order to polyadenylate the mRNA and facilitate translation (Huang et al., 2003; Martin, 2004). As mentioned above, knockdown of the *mvp* gene by RNAi decreased Aurora-A expression levels and local translational activity in neurites. These results suggest that the vault complex may function as a transporter for both mRNAs translated in dendrites and Aurora-A, which are both involved in the regulation of local translation. In fact, the vault complex was found to bind to the dendritic transport motor protein KIF5 (Hirokawa, 2006; Wakatsuki et al., 2021b), suggesting that the vault complex is transported in dendrites as cargo of KIF5.

**FIGURE 1:**
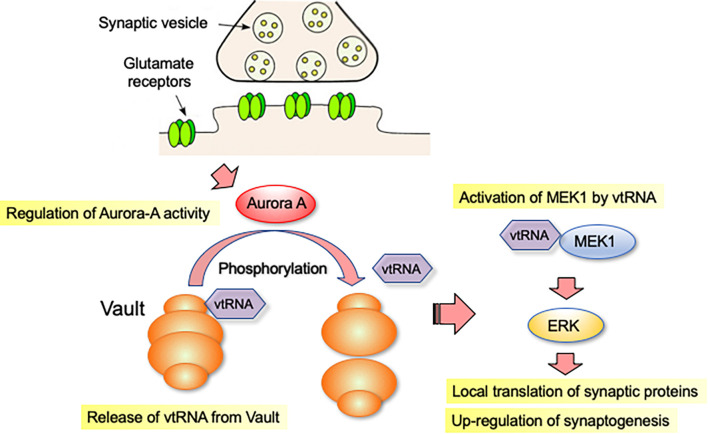
vtRNA functions as a putative riboregulator of synaptogenesis. vtRNA is transported to the distal regions of the neurites as part of the vault complex. vtRNA is released from the vault complex in the neurite by a mitotic kinase Aurora-A-dependent phosphorylation of MVP, a major protein component of the vault complex. vtRNA functions as a putative riboregulator of synaptogenesis. vtRNA binds to and activates MEK1, thereby enhancing MEK1-mediated ERK activation in neurites. Activation of ERK signaling promotes local translation of synaptic proteins, which accelerates synapse formation.

The vault complex is not a stable structure and is known to change shape dynamically (Kickhoefer et al., 1998). To correlate the activity of Aurora-A with the structural integrity of the Vault complex, we examined the biochemical behavior of the vault complex in neurons overexpressing a constitutively active form of Aurora-A. The fully assembled vault complex can be separated from incomplete/dissociated ones by high-speed centrifugation (Berger et al., 2009). Using this method, we predicted that overexpression of a constitutively active form of Aurora-A would destabilize the assembled structure of the vault complex. Furthermore, we found that mvtRNA is released from the vault complex, when the vault structure is destabilized. To understand the function of released mvtRNA, we examined how it affected ERK activity-dependent synaptogenesis. In neurites with RNAi-mediated downregulation of mvp or antisense oligonucleotide-mediated downregulation of mvtRNA, we observed reduced ERK activation, local translation and synaptic formation (Wakatsuki et al., 2021b). These results suggest that mvtRNA acts on ERK activity to potentiate local translation in neurites and positively regulate synaptogenesis (Figure 1).

**FIGURE 2:**
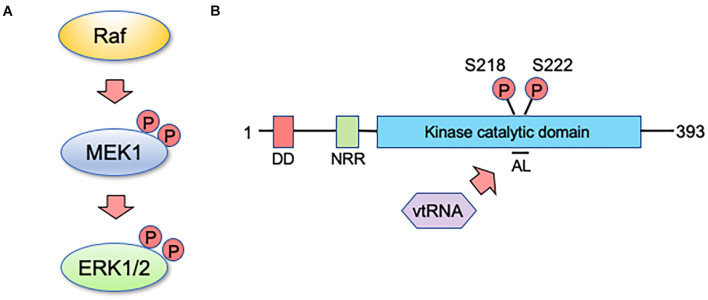
vtRNA functions as a regulator of MEK activity. **(A)** MEK1 is a core component of the MAP kinase signaling pathway, playing a key role in transmitting signals from active Ras to ERK. MEK1 is activated when it is dually phosphorylated by an upstream kinase such as Raf on two specific serine residues within its activation sequence. **(B)** Human MEK1 encodes a protein kinase of 393 amino acids. The docking domain (DD) for ERK1 and ERK2 is shown in red and the amino-terminal negative regulatory region (NRR) is shown in green. The kinase catalytic domain is shown in blue and includes the activation loop (AL) with specified sites for activating phosphorylation by Raf kinase. vtRNA appears to inhibit the interaction of the AL with the catalytic domain, and thereby enhances the kinase activity of MEK1 (see text for details).

So how does mvtRNA control ERK activity? Although there are not many reports dealing with physiological roles of vtRNA thus far, several have suggested that vtRNAs directly bind to their target proteins to regulate their functions (Koromilas et al., 1992; Meurs et al., 1993). In our analysis, we found that mvtRNA binds to MEK, thereby enhancing its ability to phosphorylate ERK (Wakatsuki et al., 2021b). Extensive knowledge has been accumulated about the relationship between molecular structure and MEK activity; and the changes in molecular structure during the MEK activating process have been investigated in detail. When MEK is inactive, the activation loop (AL) interacts with residues outside the kinase domain (e.g., negative regulatory region) and is tightly wrapped in a catalytic pocket to maintain low MEK basal activity (Ordan et al., 2018). There are two serine residues in the AL that are important for MEK activity control. Their phosphorylation by a MEK activating kinase such as Raf kinase changes the structure and activates MEK (Figure 2A; Morrison and Davis, 2003). Thus, MEK activity is considered to be altered not only by modification of kinase domain residues but also by structural changes in the AL. This may suggest the possibility that mvtRNA binding to MEK alters its molecular structure and enhances its activity (Figure 2B). However, because MEK lacks previously known RNA-binding motifs, it is not possible at this time to determine the mode of binding of both molecules. In the future, it will be necessary to clarify the binding mode between mvtRNA and MEK by methods such as the high-throughput sequencing of RNAs isolated by cross-linking immunoprecipitation.

Some of the recent reports suggest that the involvement of vtRNA1-1 in the regulation of ERK pathway may be complexed. Bracher et al. (2020) demonstrated that vtRNA1-1 loss leads to increased activation of ERK pathway in human cancer cells. Since vtRNA1-1-null conditions were used there, it is not clear whether vtRNA1-1 could inactivate ERK pathway in some contexts, but this report certainly suggests that different physiological/pathological conditions in different cell types add further complexity to the vtRNA-mediated regulation of subcellular signaling.

## Regulation of Local Translation in Dendrites and Synaptogenesis by Non-Coding RNAs

There are four vtRNA genes (hvtRNA1-1, hvtRNA1-2, hvtRNA1-3, and hvtRNA2-1) in the human genome. There are not much data on how the different nucleotide sequences of these variants influence their functions. hvtRNA2-1 differs widely from other hvtRNA1 variants except for two identical sequence blocks for Pol III transcription (Nandy et al., 2009; Stadler et al., 2009). It was previously reported that hvtRNA2-1 has no physical contact with the vault complex (Lee et al., 2011). Our results also showed that hvtRNA1-1 promotes synapse formation, whereas hvtRNA2-1 does not (Wakatsuki et al., 2021a). Amort et al. (2015) reported that in an Epstein-Barr virus-induced apoptosis model, the protective effect of only hvtRNA1-1 in the hvtRNA paralog requires a centrally located sequence, but the detailed mechanism has not yet been elucidated. Analysis of the nucleotide sequence required for the variant-specific functions of vtRNAs exemplified in these reports may lead to mechanistic understanding of the molecular basis of the variant-specific functions of vtRNAs.

Translation of proteins consists of several steps, including initiation and elongation. The initiation reaction is complex and closely regulated by multiple initiation factors, called the elongation initiation factors (eIFs; Sonenberg and Hinnebusch, 2009; Jackson et al., 2010). mTOR activity regulates the precise initiation of translation by phosphorylating eIF either directly or through the S6 kinase. CaMK2A is known to regulate the translational elongation. This kinase activation is initiated in response to neurotransmitter binding to cell surface receptors. For example, mTORC1 activity is regulated by PI3K-AKT and ERK, which are activated by NMDA receptors, etc. (Sosanya et al., 2013). Since the vault complex and Aurora-A are present at excitatory glutamatergic synapses, Aurora-A activity may be regulated via glutamate receptors (Figure 1). Interestingly, we observed that mvtRNA regulates ERK activity but has little effect on AKT activity (Wakatsuki et al., 2021b). These results suggest that vtRNAs may be involved in specific subcellular signaling elicited by neurotransmitter receptors. In the future, it will be necessary to clarify the synapse formation mechanism regulated by the Aurora-A-Vault complex in comparison with other kinase signaling pathways.

Few reports have shown that ncRNAs act locally and are directly involved in the regulation of neuron-specific events such as synaptogenesis. Among ncRNAs, miRNAs are specifically concentrated at dendrites and synapses, and it has been suggested that mature miRNAs may be generated at synapses from miRNA precursors (Kosik, 2006; Bicker et al., 2013). Translation of several different mRNAs is regulated by a group of miRNAs specifically present at synapses. One of the abundant miRNAs in dendrites, miR-26a regulates microtubule assembly via inhibition of Map2 mRNA translation in response to synaptic activation (Kye et al., 2007). Brain-specific miR-134 inhibits mRNA translation of LIMK1, which promotes dendritic spine formation at silent synapses (Schratt et al., 2006; Bicker et al., 2014). Thus, miRNAs regulate synaptogenesis through mRNA-targeted translational control, a mechanism quite different from that of vtRNA.

## Regulation of Kinase Activity by Non-Coding RNAs

Some ncRNAs act directly on kinases to regulate their activity. For example, neighbor of BRCA1 gene 2 is a long-chain ncRNA that is induced by cellular energy depletion and binds to the kinase domain of AMPK to increase its activity (Liu et al., 2016). LINK-A, identified as a long-chain ncRNA with a high affinity for the plasma membrane, binds to both the phosphatidylinositol 3,4,5-trisphosphate and the pleckstrin homology domain of AKT, and promotes their interaction to up-regulate AKT activity (Lin et al., 2017). In these examples, long-chain ncRNAs have been shown to bind directly to kinases, but it is not well understood how the ncRNA binding affects the activity of the kinases. With regard to vtRNAs, it has been reported that hvtRNA2-1 directly acts on protein kinase RNA-activated (PKR) to regulate its activity (García et al., 2007). PKR is a known interferon-induced, double-stranded RNA (dsRNA)-domain kinase and plays an important role in cellular proliferation and apoptosis (Gal-Ben-Ari et al., 2018). Previous reports suggest that PKR is a tumor suppressor (Koromilas et al., 1992; Meurs et al., 1993). Inhibition of hvtRNA2-1 activates PKR and its downstream pathways, resulting in impaired cellular proliferation. Therefore, hvtRNA2-1 is thought to be a PKR regulator, and its tumor-suppressive effect has attracted attention. Because PKR has a distinct RNA-binding motif and is primarily a kinase activated by dsRNA, hvtRNA2-1 may regulate PKR activity in a way similar to that by dsRNA. Our results show that MEK is a RNA-binding protein whose kinase activity is regulated by mvtRNA, even though MEK lacks a typical RNA-binding motif. Recent studies have identified the RNA-binding capacity of many proteins which have not previously been identified as RNA-binding proteins (Hentze et al., 2018). While MEK does not have a typical RNA-binding motif, mvtRNA may also directly bind to MEK to regulate its activity. It is necessary to clarify how the mvtRNA binding affects the molecular structure of MEK or alters the affinity of MEK to ERK to activate MAPK signaling.

## Conclusion and Future Directions

Our recent findings reveal a novel regulatory mechanism of synapse formation by the small non-coding vtRNA. Our results suggest that phosphorylation of MVP by Aurora-A activity triggers the release of mvtRNA from the vault complex. Remaining questions include detailed mechanisms by which Aurora-A dependent phosphorylation of MVP affects the structure of the vault protein complex, and by which vtRNA regulates MEK activity. For the former, we have thus far identified several serine residues in MVP whose phosphorylation prevents vault complex formation (unpublished observations). It is important to clarify how the phosphorylation of these serine residues affects the molecular structure of MVP and the release of vtRNAs from the vault complex to uncover the entire mechanism. No definitive experimental evidence has yet been obtained to show where the Aurora-A-vault complex regulates the MAPK signaling in the neurite. Knowing where the interaction between mvtRNA and MEK1 occurs in dendrites is important when considering the physiological significance of the vtRNA-dependent MEK activation mechanism. In addition to the analysis of the regulatory mechanism, other approaches may be needed to address this issue, including observation of phosphorylated ERK expression in dendrites using super-resolution microscopy.

The genes encoding MVP and ERK1 are located in the 16p11.2 region of the human chromosome. The microdeletion of this region is known to increase susceptibility to ASD (Horev et al., 2011; Ouellette et al., 2020; Rein and Yan, 2020). This suggests the critical roles of MVP and ERK1 in physiological development of the neuronal network. While a number of different factors/molecules are postulated in the pathogenesis of neurodevelopmental disorders including ASD, most such factors/molecules are involved in synapse formation during the developmental stage (Gilbert and Man, 2017; Guang et al., 2018). Thus, detailed understanding of the synapse formation mechanism is likely connected to further elucidation of the pathogenesis of neurodevelopmental disorders. A better understanding of the signaling in which the Aurora-A-vault complex is involved may lead to the development of new therapeutic strategies against such disorders.

## Data Availability Statement

The original contributions presented in the study are included in the article/supplementary material; further inquiries can be directed to the corresponding author/s.

## Author Contributions

SW developed these perspectives under the supervision of TA. SW drafted the manuscript and TA provided critical revisions. Both authors contributed to the article and approved the submitted version.

## Conflict of Interest

The authors declare that the research was conducted in the absence of any commercial or financial relationships that could be construed as a potential conflict of interest.

## Publisher’s Note

All claims expressed in this article are solely those of the authors and do not necessarily represent those of their affiliated organizations, or those of the publisher, the editors and the reviewers. Any product that may be evaluated in this article, or claim that may be made by its manufacturer, is not guaranteed or endorsed by the publisher.
